# Performance enhancement by vertical morphology alteration of the active layer in organic solar cells

**DOI:** 10.1039/c7ra13219k

**Published:** 2018-02-09

**Authors:** Sheng Bi, Zhongliang Ouyang, Qinglei Guo, Chengming Jiang

**Affiliations:** Key Laboratory for Precision and Non-traditional Machining Technology of the Ministry of Education, Dalian University of Technology Dalian 116024 P. R. China jiangcm@dlut.edu.cn; Institute of Photoelectric Nanoscience and Nanotechnology, Dalian University of Technology Dalian 116024 P. R. China; Department of Electrical and Computer Engineering, Center for Materials for Information Technology, The University of Alabama Box# 870209 Tuscaloosa AL 35487 USA; Department of Material Science and Engineering, Frederick Seitz Material Research Laboratory, University of Illinois at Urbana-Champaign Urbana IL 61801 USA

## Abstract

Bulk heterojunction organic solar cells (OSCs) have attracted worldwide attention due to their great potential as a green, flexible and low-cost renewable energy source. A vertical configuration in the active layer due to the aggregation of donor and acceptor molecules and the influence on the performance of OSCs deserve an in-depth study. In this study, five different vertical configurations of the active layer in OSCs were built up. The absorbance and indexes of the devices were theoretically analyzed. It was found that the configuration with the donor and acceptor molecules distributed equally exhibits the highest power conversion efficiency, followed by the configuration with the donor closer to the anode and the acceptor closer to the cathode, which matches experimental results well. Further analyses present the recombination, resistance, quantum efficiency and current leakage of all the configurations. It is anticipated that our results will promote the better understanding and development of the OSC field.

## Abstract 2

In this work, the P3HT/PCBM system is used as a benchmark to simulate five vertical configurations which cover all possibilities of donor and acceptor aggregation in the OSC active layer. Uniform blending of donor and acceptor results in the highest PCE.

## Graphical abstract

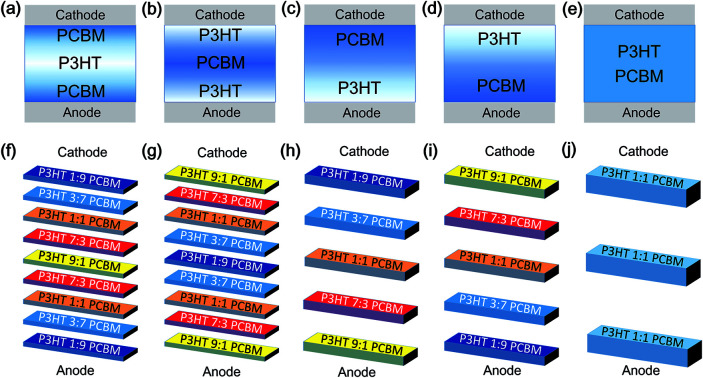

Organic solar cells (OSCs) have attracted worldwide attention due to advantages such as their low cost, flexibility, solution processability and energy saving abilities.^1–6^ The blend of polymer and fullerene morphology as produced *via* solution processing is complicated with impure phases, broad distribution of domain sizes and a vertical composition gradient.^7–11,48^ Vertical phase separation has been found to be one of the most common phenomena that exists in all OSCs.^12–16^ The distribution of donor and acceptor molecules is one of the key factors that has a great effect on charge separation and transportation. It is well known that nanoscale interpenetrating donor–acceptor network increases interfacial area, thereby enhancing exciton dissociation at the polymer–fullerene interface.

Therefore, several studies have been conducted to investigate the parameters influencing the formation of vertical phase separation, such as blend composition, post annealing, solvent evaporation rate and substrate surface energy.^17–23^ There have been substantial efforts to understand the morphology evolution in polymer:fullerene molecules out of the polymer phase, diffusion of fullerene molecules and aggregation of fullerene molecules into cluster domains. There are only three chief vertical configurations that exist in OSCs from experiment based on the way donor and acceptor molecules aggregate. Different formation of the active layers results in distinct OSC performance. Nonetheless, the mechanism of vertical aggregation of donor and acceptor molecules and the function of vertical configurations are still under debate.

In this work, the P3HT/PCBM system is used as a benchmark to simulate five different vertical configurations, which cover all the possibilities of donor and acceptor aggregation in the OSC active layer. Calculation models were established to simulate Gradient I, Gradient II, Top-down, Bottom-up and Homogeneous structures which represent different polymer:fullerene aggregations in the active layer of a regular structure organic solar cells. In these models, we perform thorough, systematic research on the optical properties of active layers and indexes of the device in the structure. The performance of these five configurations is exhibited by analysis of the open circuit voltage (*V*_oc_), short circuit current (*I*_sc_), fill factor (FF) and power conversion efficiency (PCE). The five vertical configurations achieved in the manuscript cannot be easily fabricated experimentally. Our work gives a clear and in-depth view of devices with almost all possibilities of donor and acceptor aggregation. The experimental results act as proof of the calculations. This gives an easy and accurate reference for deciding the vertical formation in organic solar cells. We anticipate that our findings will catalyze the development of these devices and push efficiency improvements.

Semiconducting emissive thin film optical simulators (SETFOSs),^5,24,25^ developed by Fluxim AG, are employed to concretely reveal charge distribution inside PSCs. As schematically described in Fig. 1(a)–(e), it was found from experiment that the vertical configuration of the P3HT and PCBM blend layer can be separated into five different cases: P3HT or PCBM aggregates in the middle sandwiched by PCBM or P3HT aggregation, P3HT or PCBM configurations with Top-down aggregation, and P3HT/PCBM mixed uniformly. In order to simulate this experiment, models were built up to fit these five situations in Fig. 1(f)–(j), assuming that the top electrode is the cathode while the bottom electrode is the anode. The simulation was composed of five different concentration ratios of P3HT and PCBM layers: 1 : 9, 3 : 7, 1 : 1, 7 : 3 and 9 : 1. In the sandwich structure, a P3HT 1 : 9 PCBM layer was simulated at the top to represent dramatic PCBM aggregation. As the concentration of PCBM decreases and that of P3HT increases in the middle, P3HT 3 : 7 PCBM, P3HT 1 : 1 PCBM and P3HT 7 : 3 PCBM were used in series to serve as the gradient concentration of PCBM. P3HT 9 : 1 PCBM was introduced at the middle to represent a P3HT-rich layer, according to experiment. An increased concentration of PCBM, P3HT 7 : 3 PCBM, P3HT 1 : 1 PCBM and P3HT 3 : 7 PCBM was created subsequently, again with the concentration of P3HT decreased. Finally, P3HT 1 : 9 PCBM was used as the PCBM-rich layer at the bottom. In the Gradient II configuration, the distribution of P3HT-rich and PCBM-rich is the opposite. P3HT 1 : 9 PCBM is set up in the middle while P3HT 9 : 1 PCBM is on both the top and bottom. The vertical configuration is dramatically changed in the Top-down configuration. At the very top, the P3HT 1 : 9 PCBM layer is the PCBM-rich layer. The gradient concentration of P3HT 3 : 7 PCBM, P3HT 1 : 1 PCBM and P3HT 7 : 3 PCBM was subsequently created from top to bottom. P3HT 9 : 1 PCBM at the bottom demonstrated severe P3HT aggregation, and the distribution was the opposite in the Bottom-up profile, where the concentration of PCBM gradually increases from top to bottom while that of P3HT went in the other direction. A ratio of 1 : 1 between P3HT and PCBM was used as the Homogeneous structure. It is worth noting that the total thicknesses of the active layer for all the structures is the same.

**Fig. 1:**
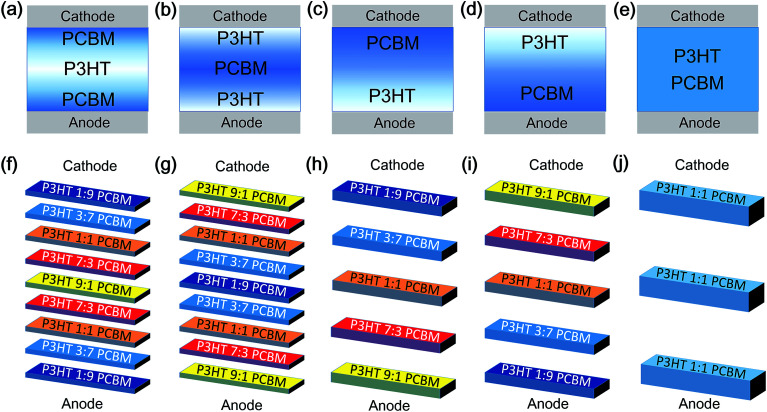
The schematic of (a)–(e) experimental conditions of polymer:fullerene aggregation and simulation models chosen accordingly as (f) Gradient I, (g) Gradient II, (h) Top-down, (i) Bottom-up, and (j) Homogeneous configurations in the regular structure of P3HT/PCBM organic solar cells.

The simulation parameters used to reproduce the measurements are summarized in Table 1. The work function of the anode and cathode is 5.0 eV and 4.3 eV, respectively. The dielectric constant varies with the ratio between P3HT and PCBM. The dielectric constant for pure P3HT is 3 while that for pure PCBM is 3.9, and the dielectric constant changes linearly with the content.^26–31^ The parameters of mobility and Langevin recombination efficiency and optical charge generation efficiency for each P3HT/PCBM ratio are reasonably set. Since the acceptor (PCBM) carries holes, an increasing amount of acceptor leads to easier electron transportation, which results in the electron mobility improvement from P3HT 9 : 1 PCBM to P3HT 1 : 9 PCBM. The same goes for holes. The decreasing amount of donor (P3HT) produces the reduction in hole mobility, which matches the conclusions from experiment well.^32,33^ Also, according to previous experiments,^34–37^ the proper amount of P3HT and PCBM molecules (a fair amount of P3HT and PCBM) in the blend enormously stimulates charge separation and transportation and thus leads to a high optical charge generation efficiency and low Langevin recombination efficiency. In P3HT 9 : 1 PCBM and P3HT 1 : 9 PCBM, high Langevin recombination efficiency originates from a smaller interface, where electron and hole pairs separate.^34–37^ And P3HT 9 : 1 PCBM generates charge better than P3HT 1 : 9 PCBM because of having more P3HT molecules helps with generation. The values for electron/hole mobility, Langevin recombination efficiency and optical charge generation efficiency were obtained from individual P3HT 9 : 1 PCBM, P3HT 7 : 3 PCBM, P3HT 1 : 1 PCBM, P3HT 3 : 7 PCBM and P3HT 1 : 9 PCBM OSC devices and match those from experimental results in the literature well.^38–42^ In the simulation of the multi-layer structure, these parameters were no longer changed.

**Table 1:** Parameters used in calculations for different ratios of P3HT and PCBM

Parameter	P3HT 9 : 1 PCBM	P3HT 7 : 3 PCBM	P3HT 1 : 1 PCBM	P3HT 3 : 7 PCBM	P3HT 1 : 9 PCBM
Electron mobility (m^2^ V^−1^ s^−1^)	2 × 10^−5^	2 × 10^−4^	1 × 10^−3^	1.5 × 10^−2^	4 × 10^−2^
Hole mobility (m^2^ V^−1^ s^−1^)	7 × 10^−3^	2 × 10^−3^	7 × 10^−4^	2.5 × 10^−4^	5 × 10^−5^
Langevin recombination efficiency	0.05	0.15	0.2	0.12	0.05
Optical charge generation efficiency	0.2	0.6	0.8	0.52	0.1
Dielectric constant	3.09	3.27	3.45	3.63	3.81

For the experiment, P3HT (50 kDa) was commercially available from Rieke Metal Inc., and PCBM was purchased from NanoC and used as received. A 30 nm-thick poly(3,4-ethylenedioxy thiophene):poly(styrene sulfonate) (PEDOT:PSS) anode buffer layer, purchased from HC Stark, was spin-coated on top of the precleaned ITO substrate, which was treated with soap, acetone and isopropyl alcohol for 15 min and UV ozone for 20 min, then dried at 140 °C for 10 min in air. The P3HT–PCBM (1 : 0.7 wt) was dissolved in 1,2-dichlorobenzene (DCB) and the resulting solution (concentration, 20 mg ml^−1^) was deposited at a speed of 900 rpm for 40 s on top of the PEDOT:PSS layer. Then the entire device was put in a vacuum oven and annealed at 140 °C for 20 min. An 80 nm Al layer was subsequently thermally evaporated at a pressure of 3 × 10^−6^ Torr. Current–voltage (*I*–*V*) characterization of the polymer photovoltaic cells was conducted using a computer-controlled measurement unit from Newport under AM1.5G illumination, 100 mW cm^−2^.

Fig. 2(a) shows the current density *versus* voltage (*J*–*V*) characteristics from a simulation of the regular OSC structure with different vertical configurations. The power-conversion efficiencies (PCEs) extracted from these *J*–*V* curves are summarized in Fig. 2(b). From the *J*–*V* curve, both Top-down and Homogeneous structures tend to have comparably square-shaped curves because of the easy charge carrier transportation, which leads to a relatively high fill factor. The shape of the curves in the Gradient I and Gradient II configurations tends to be S-shaped, which leads to a low fill factor. The open circuit voltage in the Bottom-up configuration is obviously much smaller than those in all the other configurations. The Homogeneous configuration tends to have the highest current density. The PCEs are 1.15%, 1.89%, 3.08%, 1.53% and 3.96% for the Gradient I, Gradient II, Top-down, Bottom-up and Homogeneous configurations, respectively.

**Fig. 2:**
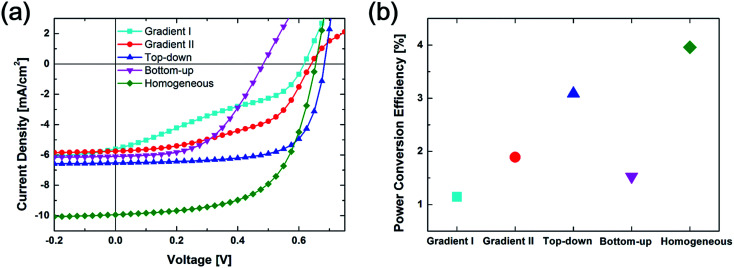
(a) *J*–*V* curve and (b) power conversion efficiency from Gradient I, Gradient II, Top-down, Bottom-up and Homogeneous configurations in simulation.

The absorbance of a solar cell with different vertical compositions has been studied. As illustrated in Fig. 3(a), the absorbance of the different structures exhibits a similar trend, first dropping to 450 nm and then rising to 600 nm. Beyond 600 nm, the absorption decreases drastically with increasing wavelength. The Bottom-up configuration has the lowest overall absorbance, and there is no significant difference in absorption for the other four structures. This behavior can be explained using the complex refractive index *n̄* = *n* + i*κ*. The imaginary part, *κ*, which is also called the extinction coefficient, plays a key role in the light absorbing process. The intensity of the electromagnetic wave that propagates through a material is proportional to exp(−4π*κd*/*λ*), where *d* and *λ* are the depth into the material and wavelength in the vacuum, respectively. Thus, light passing through a material with larger *κ* attenuates faster. In other words, light experiences stronger absorption across materials with higher *κ* values. Fig. 3(b) shows the wavelength dependence of extinction coefficients and the normalized absorbance for the P3HT 1 : 1 PCBM layer. It can be seen that the trend of extinction coefficients has been mimicked by the spectral absorbance. The slight difference between the absorbance for different configurations is probably a result of the reflection occurring at the interfaces between layers.

**Fig. 3:**
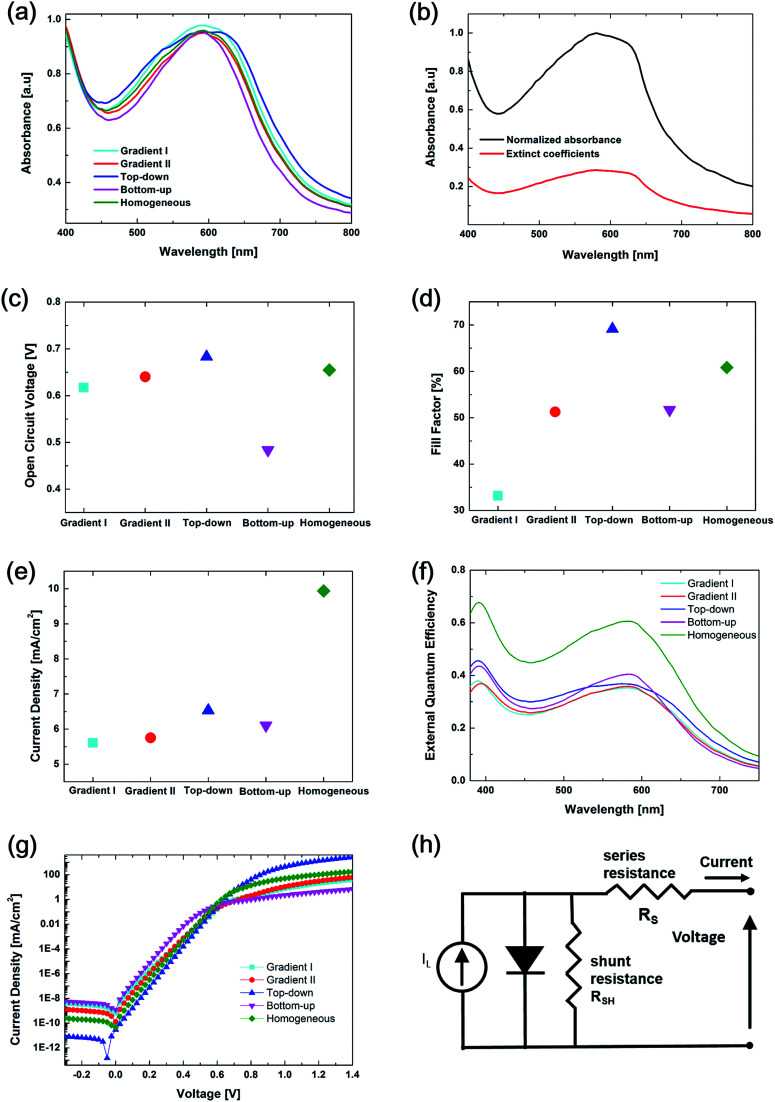
(a) Absorbance and (b) extinction coefficients of Gradient I, Gradient II, Top-down, Bottom-up and Homogeneous configurations in simulation. (c) Open circuit voltage, (d) fill factor, (e) current density, (f) external quantum efficiency, (g) dark current density, and (h) equivalent circuit of Gradient I, Gradient II, Top-down, Bottom-up and Homogeneous configurations in simulation.

Open circuit voltage (*V*_oc_) variations based on different vertical configurations are shown in Fig. 3(c). The Top-down configuration has the highest value compared to the lowest value from the Bottom-up configuration. The values from Gradient I, Gradient II and Homogeneous configurations also vary greatly. The general expression is shown in eqn (1),^43^1
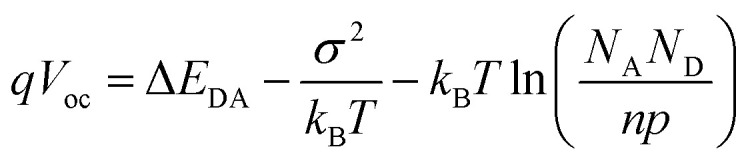
where Δ*E*_DA_ is the effective bandgap. *N*_D/A_ represents the total density of hole/electron states and *σ* is the width of the Gaussian DOS. The three terms on the right hand side of the equation are the effective bandgap, disorder-induced *V*_oc_ loss and the carrier recombination induced *V*_oc_ loss. In this experiment, the HOMO and LUMO of P3HT/PCBM are kept unchanged all the time, thus Δ*E*_DA_ is constant. The main reason for the cutback of *V*_oc_ comes from the latter two factors. Charge carriers are more likely to get trapped and recombined because of the vertical configuration in the Bottom-up configuration, which leads to a reduction of the open circuit voltage. In contrast, the Top-down configuration promotes electron and hole transportation all the time, and thus has the best open circuit voltage. The Gradient I configuration is the combination of Top-down and Bottom-up configurations. Therefore, the bottom half blocks charge carriers while the top half promotes transportation, resulting in the value of *V*_oc_ being between that of the Top-down and Bottom-up configurations. The Gradient II configuration is in the same situation but P3HT is closer to the anode, allowing more sunlight to go through the device and thus generating more charge carriers, leading to a slightly higher *V*_oc_ than that in the Gradient I configuration. In the Homogeneous configuration, equivalence all across the layers results in neither heavy trapping nor improved promotion.

Fill factor variation, as shown in Fig. 3(d), can be expressed by eqn (2) and (3),^44,45^2
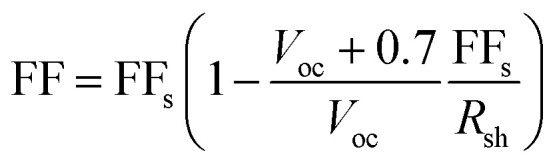
3
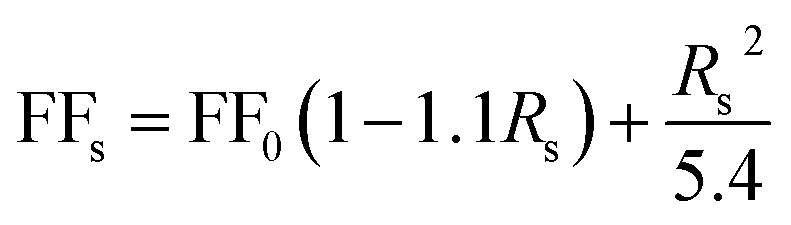
where FF_0_ is the fill factor of an ideal solar cell. *R*_sh_ and *R*_s_ are the shunt resistance and series resistance, respectively. Series resistance is believed to originate from the electrodes, bulk resistance of the active layer and contact resistance between the active layers. Shunt resistance derives from manufacturing defects such as pinholes in the cell and a variety of current leakage. Resistance can be derived from dark current curves which are also extracted from simulation, as shown in Fig. 3(g). Based on Shockley’s p–n junction model, the current density can be written as:^46^4
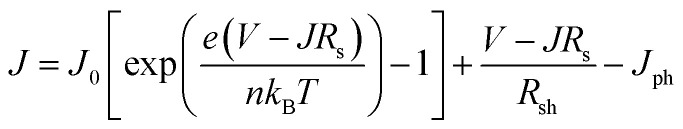
where *J*_0_ is the reverse bias saturation current density, *e* is the elementary charge, *R*_s_ is the series resistance, *n* is the diode ideality factor, *k*_B_ is Boltzmann’s constant, *T* is temperature, *J*_ph_ is photocurrent and *R*_sh_ is the shunt resistance. The first part of the equation represents the recombination current. It accounts for how a solar cell acts as a diode in the dark. The second part represents the shunt current, which refers to the cell leakage due to sources such as pinholes that enable parasitic current to move directly from one electrode to the other.^46,47^ From the solar cell equivalent circuit in Fig. 3(h), a larger shunt resistance (*R*_sh_) and smaller series resistance (*R*_s_) give the best performance of the device. In a dark *J*–*V* curve, the slope of the curve where the voltage is small represents *R*_sh_, while *R*_s_ dominates where the voltage is large. From Fig. 3(g), when the voltage is above 0.6 V, there is a distinct slope of curves from different configurations. The Top-down and Homogeneous configurations have larger slopes, meaning that the *R*_s_ in these devices is small. The Bottom-up configuration has the smallest slope, which indicates that the *R*_s_ is the largest. This is one of the factors that leads to poor performance. Also, the dark current at zero voltage represents current leakage. Top-down configuration obviously undergoes the least current leakage, thus its *I*_sc_ is the highest among the five.

Current density variation is shown in Fig. 3(e). It depends on the amount of charge carrier generation and the resistance in the device. The External Quantum Efficiency (EQE), shown in Fig. 3(f), reflects the ratio between the number of generated electron–holes by excitons in the device and the number of incident photons. The Homogeneous configuration possesses the highest EQE value, which means a lot more electrons and holes in the device are generated by incident photons. As a result, a noticeably high current density occurs in the Homogeneous configuration. The EQE values of the Top-down and Bottom-up configurations are relatively similar and those of Gradient I and Gradient II are the lowest. The current densities of Gradient I, Gradient II and Bottom-up configurations have minor differences under the function of EQE, resistance and current leakage.

In order to calculate the goodness of fit between the experimental and computational results, a *J*–*V* curve comparison is carried out, as shown in Fig. 4. In Fig. 4(a), the *J*–*V* curve from the experiment with 3.1% PCE is presented. *J*–*V* curves from various vertical configurations are compared with those from experiment. Variance between each two curves was calculated as 13.9, 8.1, 3.8, 15.2, and 2.4 for the curves between the Gradient I, Gradient II, Top-down, Bottom-up, and Homogeneous configurations and experimental values, respectively. As a result, the data for the Homogeneous configuration is the closest to experimental data followed by the Top-down vertical configuration data.

**Fig. 4:**
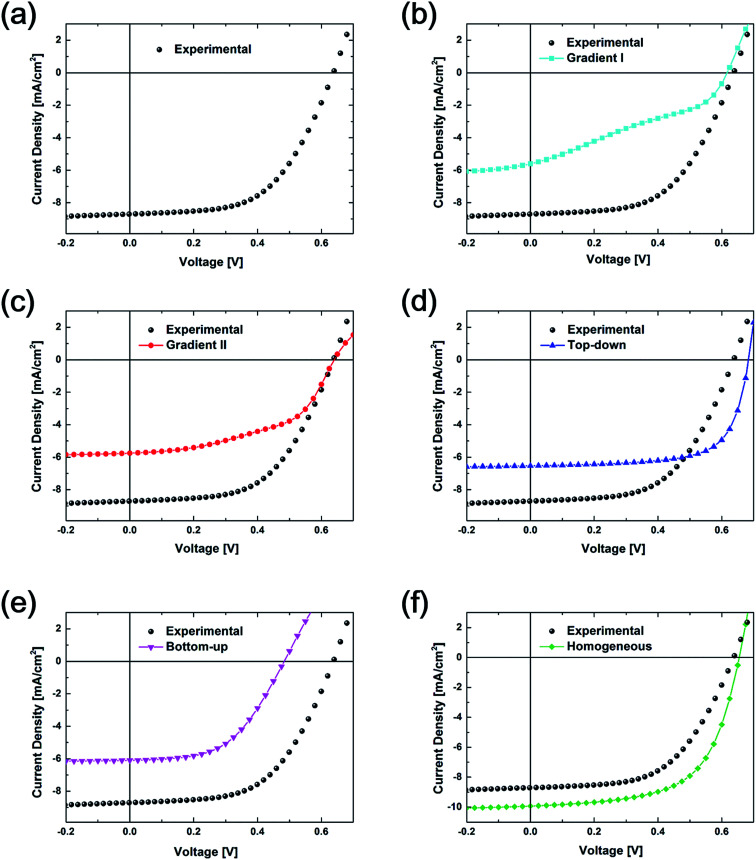
Current density *vs.* voltage curves of (a) experimental results, (b) experimental results *vs.* Gradient I, (c) experimental results *vs.* Gradient II, (d) experimental results *vs.* Top-down, (e) experimental results *vs.* Bottom-up, and (f) experimental results *vs.* Homogeneous vertical configuration from the simulation.

To conclude, five vertical configurations have been successfully simulated. The vertical configuration has a dramatic effect on the performance of OSCs. It was found that the variation of PCE in different configurations comes from the quantum efficiency, resistance in the device and the current leakage under various circumstances. Uniform blending of the donor and acceptor results in the highest PCE. The acceptor being closer to the cathode and the donor being closer to the anode also gives a relatively high PCE. By comparing the variance between simulation and experimental data, the Homogeneous configuration has the most similar data to that from experiment. This finding emphasizes that a difference in vertical configuration can lead to a superior overall device performance and provides guidance for vertical configuration design in experiments. This gives an easy and accurate reference for deciding the vertical formation in organic solar cells. We anticipate that our findings will catalyze the development of OSCs and promote a better understanding in the field.

## Conflicts of interest

There are no conflicts to declare.

## Supplementary Material
